# Experimental postoperative ileus: is Th2 immune response involved?

**DOI:** 10.7150/ijms.59354

**Published:** 2021-06-16

**Authors:** Sisi Lin, Florian Kühn, Tobias S. Schiergens, Andrey A. Zamyatnin, Orkhan Isayev, Eldar Gasimov, Jens Werner, Yongyu Li, Alexandr V. Bazhin

**Affiliations:** 1 Department of Pathophysiology, Institute of Digestive Disease, Tongji University School of Medicine, 200092, Shanghai, China.; 2 Department of General, Visceral, and Transplantation Surgery, University Hospital, LMU Munich, 81377, Munich, Germany.; 3 Institute of Molecular Medicine, Sechenov First Moscow State Medical University, 119991, Moscow, Russia.; 4 Department of Cell Signaling, Belozersky Institute of Physico-Chemical Biology, Lomonosov Moscow State University, 119991, Moscow, Russia.; 5 Department of Histology, Embryology and Cytology, Azerbaijan Medical University, Baku, Azerbaijan.; 6 German Cancer Consortium (DKTK), Partner Site Munich, 81377 Munich, Germany.

**Keywords:** Postoperative Ileus, Th2 Cells, ROS, Mast Cells, IgE

## Abstract

**Rationale:** Postoperative ileus (POI) is a frequent complication arising after gastrointestinal surgery but pathogenesis of POI is still not fully understood. While Th1 immune cells are implicated in POI, the involvement of Th2 cells has not yet been clarified. Given the impact of reactive oxygen species (ROS) in the regulation of Th1 and Th2 balance, we hypothesized that not only Th1 but also Th2 immune response can be involved in the development of experimental POI.

**Methods:** The intestinal transit test was performed using carbon gum arabic. Electron microscopy was employed to assess tissue morphology and the presence of immune cells. Cytokines, IgE and ROS were measured. Immune cells from Peyer's patches were analyzed by Flow Cytometry and toluidine blue staining was used for detection of mast cells. Transcriptional factors were analyzed by Western blot.

**Results:** POI is associated with an increase in both Th2 cytokines and Th2 cells. We have further demonstrated that POI induces a Th2-dependent activation of memory and non-memory B cells. This was accompanied by an increase in a number of mast cells in the colon of POI mice as well by an increased IgE and histamine plasma levels. We found that POI-induced accumulation of ROS was associated with an increased expression of the transcriptional factors HMBGI, NF-κB, and p38. This increased expression seemed to be associated with a Th2 response.

**Conclusion:** Th2 immune response can be involved in the activation of mast cells in POI, which was associated with ROS mediated activation of NF-κB and p38 MAPK signaling pathway.

## Introduction

Postoperative ileus (POI) represents one of the most frequent complications after gastrointestinal surgery, with an incidence between 10-30% 1, 2. It is characterized by a generalized hypomotility of the gastrointestinal tract, leading to postoperative malaise including pain, nausea, vomiting and food intolerance 3. POI is associated with a significantly prolonged hospital stay and increased health care expenditures, causing a relevant socio-economic burden. Minimally invasive and enhanced recovery after surgery (ERAS) are important factors for the prevention of POI 4. However, at present, there are still no targeted and thus effective therapeutics for POI. None of the routinely used prokinetic medications were able to reduce the duration of POI in available meta-analyses.

As pathogenesis and characteristics of the immune response in POI are still not completely understood, further research is needed to help identify crucial targets for POI treatment. So far, neurogenic disturbance and inflammatory response are thought to be the main mechanisms involved in the molecular pathogenesis of POI 1, 2. Immune components which include cellular counterparts of both innate and adaptive immune systems are well known to be critical factors in the pathogenesis of POI. In addition, increased levels of reactive oxygen species (ROS), which are known to influence the balance between Th1 and Th2 response, have recently been linked to POI 2. While Th1 cells are well-known to play an important role in the inflammatory process underlying POI 3-5, the exact role of Th2 cells in this disease is still not defined. Given the impact of ROS in controlling Th1 and Th2 balance, we hypothesized that not only Th1 but also Th2 immune response is crucially involved in the development of experimental POI.

## Materials and Methods

### Materials

Gum arabic, activated carbon, 2,7-dichlorofluorescein diacetate (DCFDA), toluidine blue, protease inhibitor cocktail tablets, phosphatase inhibitor cocktail tablets, MILLIPLEX MAP Mouse Cytokine/Chemokine Magnetic Bead Panel were purchased from Merck KGaA (Darmstadt, Germany). 25% Aqueous Solution Glutaraldehyde, Paraformaldehyde, Sorensen's Phosphate Buffer, 2% Aqueous Solution Osmium Tetroxide, Ethyl Alcohol, Acetone, Araldite, Dibutyl phthalate (DBP) were purchased Electron Microscopy Sciences (Hatfield, USA). Leukocyte Activation Cocktail with BD GolgiPlug™, FACS antibodies include anti-mouse I-A/I-E-BV510, IgG1-BB700, IgM-BV605, IgE-BV786, IgD-BV711, CD1d-BV421, CD5-PE, CD45-BUV395, CD19-APC, CD45R/B220-BUV496, CD45-APC-Cy7, CD3e-FITC, CD4-V450, CD8-BV510, CD25-BV605, IL-4-PE-Cy7, IFN-γ-PE, FoxP3-AF647, CD103-BUV395, F4/80-BV711, CD80-BV650, CD11b-BV510, Ly6-G-PerCP Cy5.5, PE-Ly6-C, CD45-APC-Cy7, CD11c--AF700 were purchased from BD (Heidelberg, Germany); Another FACS antibody Anti-mouse-IL-17A-BV650 was purchased from eBioscience (Frankfurt am Main, Germany). Anti-mouse HMGB1, Anti-mouse phospho-p65, anti-mouse phospho-p38, anti-mouse GAPDH and anti-rabbit IgG HRP were obtained from Cell signaling Technology (Frankfurt am Main, Germany). IgE and histamine ELISA kits were purchased from Abcam (Berlin, Germany).

### Mice

Male and female C57BL/6J wild type (WT) mice, 8-10 weeks, were purchased from Charles River Laboratories (Sulzfeld, Germany). Mice were housed in the animal facility of the Walter Brendel Centre of Experimental Medicine LMU Munich under pathogen-free and 12 h light-dark cycle conditions. Before the start of the experiments, there was a period of acclimation of 7 days. All animal procedures were performed according to local ethical guidelines and approved by the district government of Upper Bavaria (55.2-1-54-2532-95-2016).

### POI induction and Intestinal transit test

Mice were randomly assigned into 3 groups with at least 6 mice in each group: (1) Control group (con): no surgery; (2) Sham group (sham): laparotomy with intestinal exposure but without further manipulation; (3) POI group (POI): laparotomy with intestinal manipulation. POI was induced according to the method of Li et al. 6. Briefly, mice were anesthetized with an intraperitoneal application of (2.5µl/g, i.p.) Midazolam, Medetomidin and Fentanyl. To relieve pain, Novalgin (1.5µl/g, s.c.) was administrated before surgery. After midline-laparotomy, the small intestine was mobilized out of the abdominal cavity and put on a small wet gauze. Then, the small intestine was manipulated by rolling sterile wet cotton swabs along the intestine for 5 min. For sham-operated animals, the small intestine was only taken out of the abdominal cavity and put on a wet gauze for 5 min without further manipulation. After surgery, the mice received an antagonist (8.5µl/g, i.p.) which contained Atipamezol, Naloxon and Flumazenil. After 24 h of surgery, mice received a gum arabic suspension with active carbon by gavage (0.1 ml/10 g). 20 min later, the blood was collected by heart puncture under anesthesia. Then, the mice were sacrificed and the intestine was separated from the abdominal cavity for the measurement of the distance that carbon has been pushed forward and the whole length of the small intestine. The intestinal transit was calculated with equation (carbon passed distance / whole length of small intestine x 100) as described elsewhere 7. Then ileac and colonic species were evenly divided into 1cm start from the cecum and arranged for the different detection. 5 Peyer's patches (PPs) in the small intestine were collected from each mouse. For the detection of histamine and toluidine blue staining, except for 24 h, blood and colonic segments were also collected after 12 h and 48 h of surgery.

### Lymphocytes isolation and Flow Cytometry analysis

The PPs from the intestine were collected, grounded and rinsed through 40 μm mesh cell strainers filled with cell culture medium for harvesting cell suspension. The cells were counted by using a blood counting chamber and used in approximately 1x10^6^ cells/ml concentrations. For the monocyte/macrophages panel, the cells were stained with antibodies against surface markers of interests (anti-mouse CD45R-BUV496, CD103-BUV395, F4/80-BV711, CD80-BV650, CD11b-BV510, Ly6-G-PerCP Cy5.5, PE-Ly6-C, CD45-APC-Cy7, CD11c-AF700). For the B cells panel, the cells were stained with antibodies against surface markers of interests (anti-mouse I-A/I-E-BV510, IgG1-BB700, IgM-BV605, IgE-BV786, IgD-BV711, CD1d-BV421, CD5-PE, CD45-BUV395, CD19-APC, CD45R/B220-BUV496, and CD3-FITC). For the T cells panel, incubation with the following antibodies against surface markers of interests was done (anti-mouse CD45-APC-cy7, CD3-FITC, CD4-V450 and CD8-BV510). For measurement of intracellular markers (anti-mouse IL-4-PE-Cy7, IL-17A-BV650 and IFN-γ-PE), the cells were fixed, permeabilized with 1×Perm Buffer IV and subsequently stained with the above-mentioned antibodies. The staining procedure was performed in the dark. For T cell stimulation, the cells were additionally incubated with or without RMA/Iono for 6 h. After washing with the Flow Cytometry (FACS) buffer, the cells were then resuspended in 500 μl FACS buffer and measured with BD FACS Fortessa. The gating strategies in detail were present in Figure S1 and Figure S2.

### Electron and light microscopies

The material for electron microscopy was fixated in a solution containing 2% Paraformaldehyde, 2% Glutaraldehyde and 0.1% Picrin acid prepared in phosphate buffer (pH 7.4). After the postfixation procedures in 1% Osmium acid solution for 1.5 h, the Araldite-Epon blocks were prepared according to general methods accepted in electron microscopy. Ultrathin sections (50-70 nm) were obtained by the aid of Leica EM UC7 ultramicrotome (Germany) Semithin sections were stained with methylene blue, azure II and fuchsin were studied under a Primo Starlight microscope (Zeiss, Germany). The images were photographed with a Canon digital camera (Japan). Ultrathin sections were stained with a 2% saturated aqueous solution of uranyl acetate, then with a 0.2% solution of pure lead citrate in 0.1 M NaOH solution and examined under the TEM JEM-1400 (Japan) at an accelerating voltage of 80-100 kV. Digital image files were obtained with a Veleta 2k×2k CCD camera (Olympus-Soft Imaging Solutions, Münster, Germany) under the iTEM software (Olympus-SiS).

### Toluidine blue staining for mast cell

The staining of the mast cells was done as previously described 8. Briefly, fragments about 1 cm of ileum and colon specimens were stored in 10% formalin. Then, the specimens were embedded in paraffin and cut at 5 μm sections using a microtome. The sections were deparaffinized, dehydrated and finally stained with Toluidine blue (pH2.5).

### Measurement of ROS

The ROS levels in ileac and colonic tissues were detected according to Flora and colleagues using 2,7-dichlorofluorescein diacetate (DCFDA) 9. In general, 1% of tissue homogenates were prepared with a cold 40 nM Tris-HCl buffer (pH 7.4). Then the samples were further diluted with the same buffer to an end concentration of 0.25%. 40 µl of 1.25 mM DCFDA in methanol was added into one fraction of the 2 ml of the homogenate. The second fraction of this volume contained methanol only was served as a control. The samples were then incubated for 15 min at 37 ℃. The fluorescence was measured at 488/525nm (excitation/emission) using a FilterMax F3 microplate reader. Subsequently, the ratio between the fluorescence intensity of the experimental sample and that of the control sample was used to express the ROS level.

### Western blot analysis

Ileum and colon segments were lysed with 1X RIPA buffer containing protease and phosphatase inhibitor cocktails. Protein concentration was assessed with the BCA method. Western blot analysis was carried out as described previously 10. After blocking with 5% (*w*/*v*) of milk in TBST, the PVDF membranes were incubated overnight with an antibody against the protein of interest (Anti-mouse HMGB1, phospho-p65, phospho-p38 and GAPDH), followed with incubation with secondary antibodies for 1 h at room temperature. The protein bands were detected with ECL substrate using a chemiluminescent detection system, the intensity of the bands was analyzed with the ImageJ software.

### ELISA

Serum was obtained from blood collected by heart puncture and centrifugated at 4 ℃, 5,000 rpm for 15 min. IgE and histamine concentrations in serum were measured using ELISA kits mentioned above according to the manufacturer's instructions.

### LUMINEX (Bioplex) assay

Serum was obtained from blood collected by heart puncture. Measurement of serum murine anti and pro-inflammatory mediators (IFN-γ, TNF-α, IL-1β, IL-2, IL-4, IL-5, IL-6, IL-10, MCP-1, IL-12p40, IL-12p70, IL-13 and IL-17) were performed using the MILLPLEX map kit according to the manufacturer's instructions as described previously 11. The measurement was performed using a Bio-Plex® 200 system.

### Statistical analysis

The results are expressed as mean ± standard deviation (SD). Statistical analysis was performed using GraphPad Prism 7. The null hypothesis (mean values were equal) versus the alternative hypothesis (mean values were not equal) was tested by one-way ANOVA with Tukey's post-hoc test. All statistical tests were two-tailed. The significance level was α=5%.

## Results

### POI leads to a systemic increase in Th1 and Th2 cytokines

**Figure 1:**
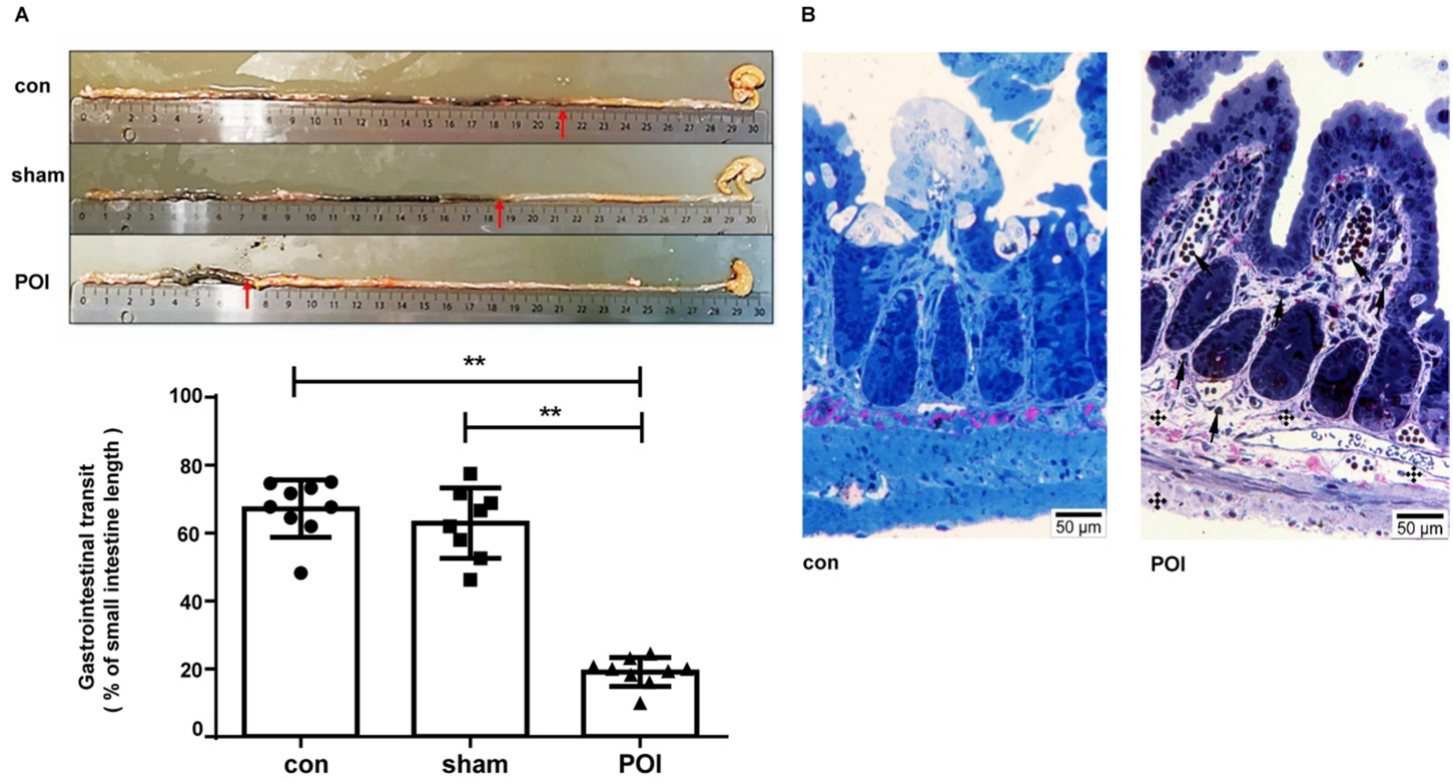
Measurement of POI. (A) Measurement of the gastrointestinal transit and (B) morphological changes with light microscopy showing changes of experimental POI such as edema and infiltration of inflammatory cells (cross: edema; arrow: lymphocyte infiltration).

**Figure 2:**
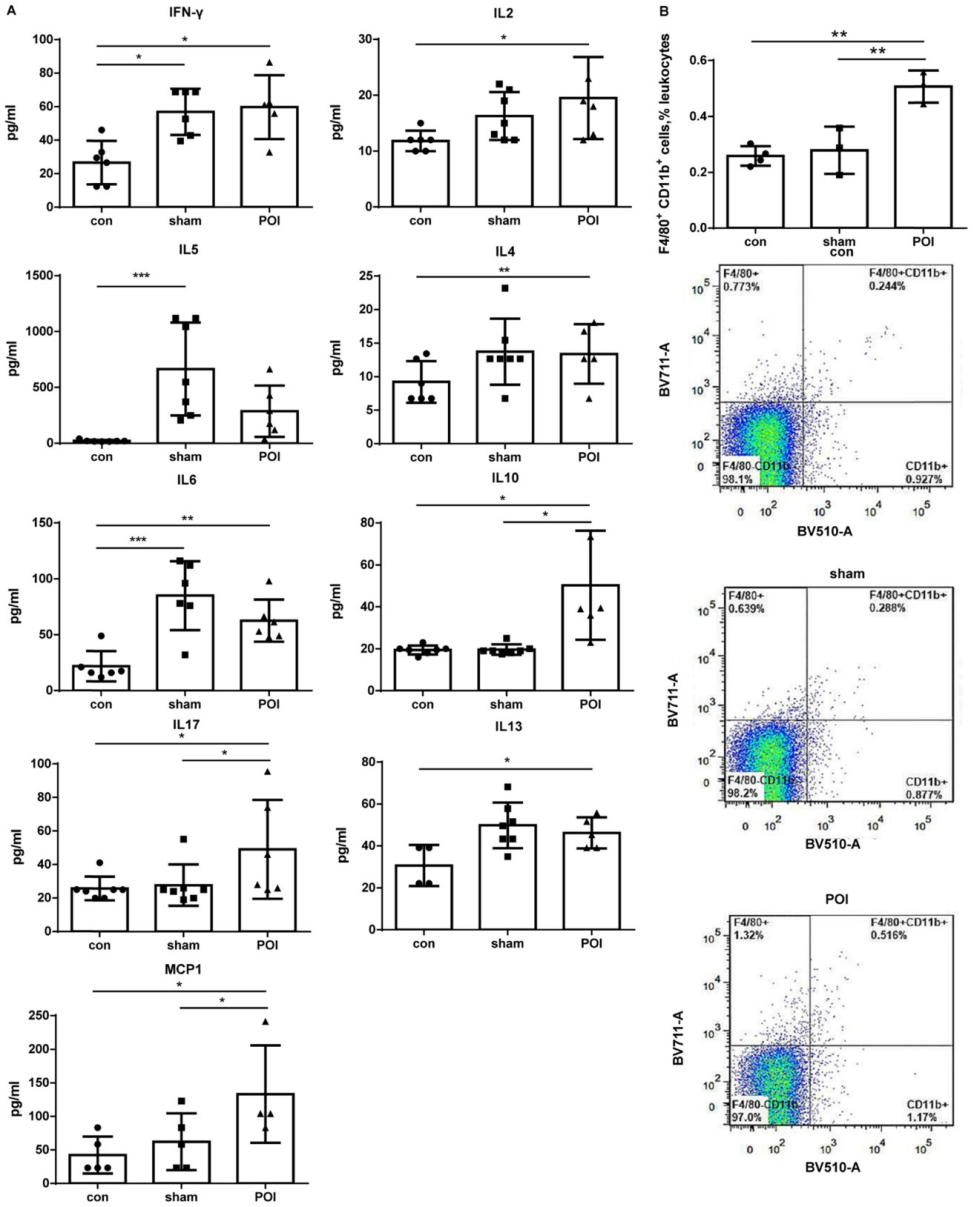
Plasma cytokine levels. (A) The Luminex analysis of cytokines in plasma was performed as described in *Materials and Methods*. Data is presented as a column bar graph with SD and analyzed with the ordinary one-way ANOVA with Tukey's post-test, * *p*<0.05, ** *p*<0.01 and *** p<0.001, n=5-7 (mice per group). (B) Accumulation of F4/80^+^CD11b^+^ in the PPs of mice from the POI group measured with FACS and presented as a bar graph (mean with SD), n=3-4 (per group), analyzed with the ordinary one-way ANOVA with the Tukey's post-test, * *p*<0.05 - upper panel, and as a representative FACS picture - lower panel.

The sham procedure led to a slight but not significant difference in the intestinal motility. The intestinal motility, measured by the carbon distance test, represents a typical read-out for POI (Figure 1A). Microscopically, pathological changes of experimental POI such as edema and infiltration of inflammatory cells were observed in POI mice (Figure 1B). Sham operation alone resulted in increased plasma levels of IFN-γ, IL-5 and IL-6 compared to control mice, whereas mice with POI possessed a significantly higher amount of IL-2, IL-10, and MCP-1 compared to sham-operated animals (Figure 2A). These findings indicated that not only Th1 immune response (IL-2) but also Th2 immune cells (IL-10) may be involved in the development of experimental POI. Therefore, we performed a profound FACS analysis of immune cells obtained from small intestinal PPs. Compared to both controls, we found a marked increase in the percentage of monocytes/macrophages in PPs obtained from POI mice (Figure 2B) as a possible source of IL-10 and MCP-1.

### POI induces Th2-dependent activation of memory B cells, non-memory B cells, secretion IgE and activation of mast cells

**Figure 3:**
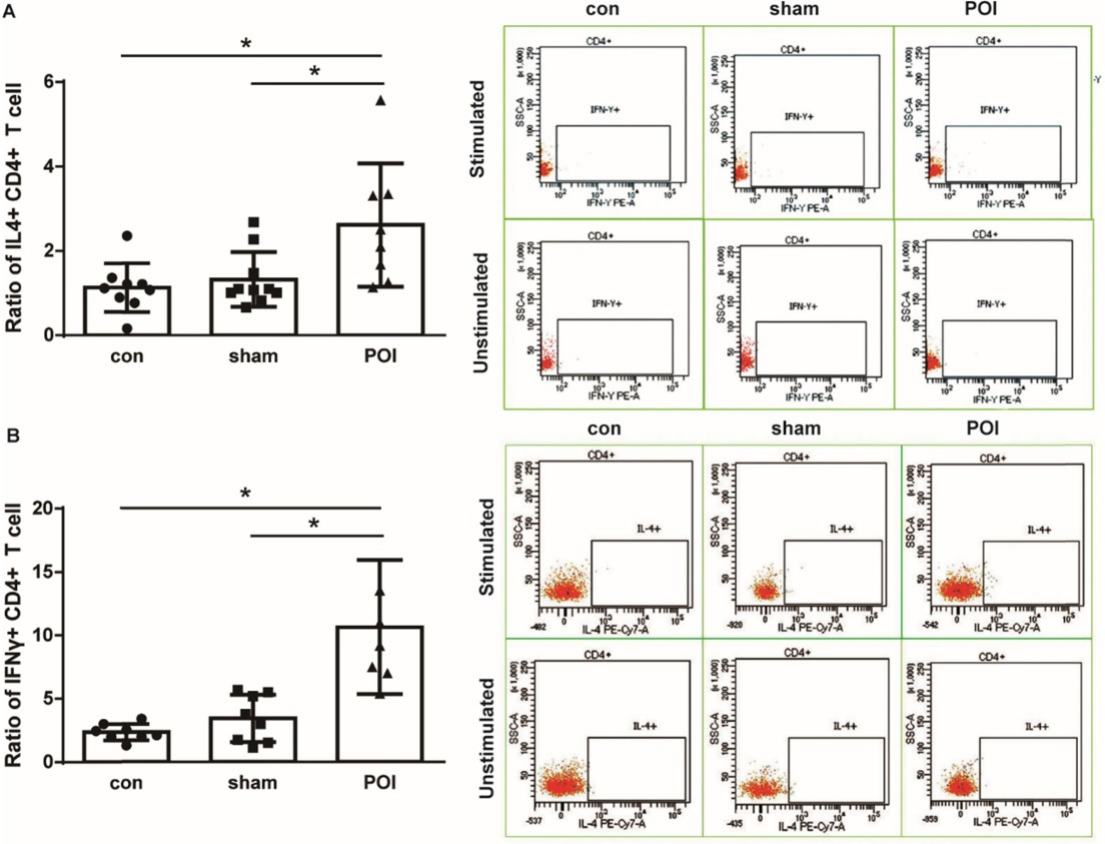
Th1 and Th2 immune response. Ratio of intracellular IFN-γ (A) and IL-4 (B) production in CD4^+^ T cells is presented as a bar graph (mean with SD), n=6-10 (per group), analyzed with the ordinary one-way ANOVA with the Tukey's post-test, * p<0.05 - left panel, and as a representative FACS picture - right panel.

POI mice showed a significant intracellular IFN-γ accumulation by CD4^+^ lymphocytes (Figure 3A). Since we detected a systemic increase in the amount of IL-10 as well as MCP-1 which are both known to be involved in the polarization of Th0 into Th2 lymphocytes, we further investigated the intracellular IL-4 production in T cells. Lymphocytes of PPs from mice were able to produce IL-4 leading to a significant increase in the intracellular concentration of the Th2-related cytokine in POI (Figure 3B). Of note, POI did not influence the intracellular IL-17 expression in the lymphocytes from PPs (data not shown).

**Figure 4:**
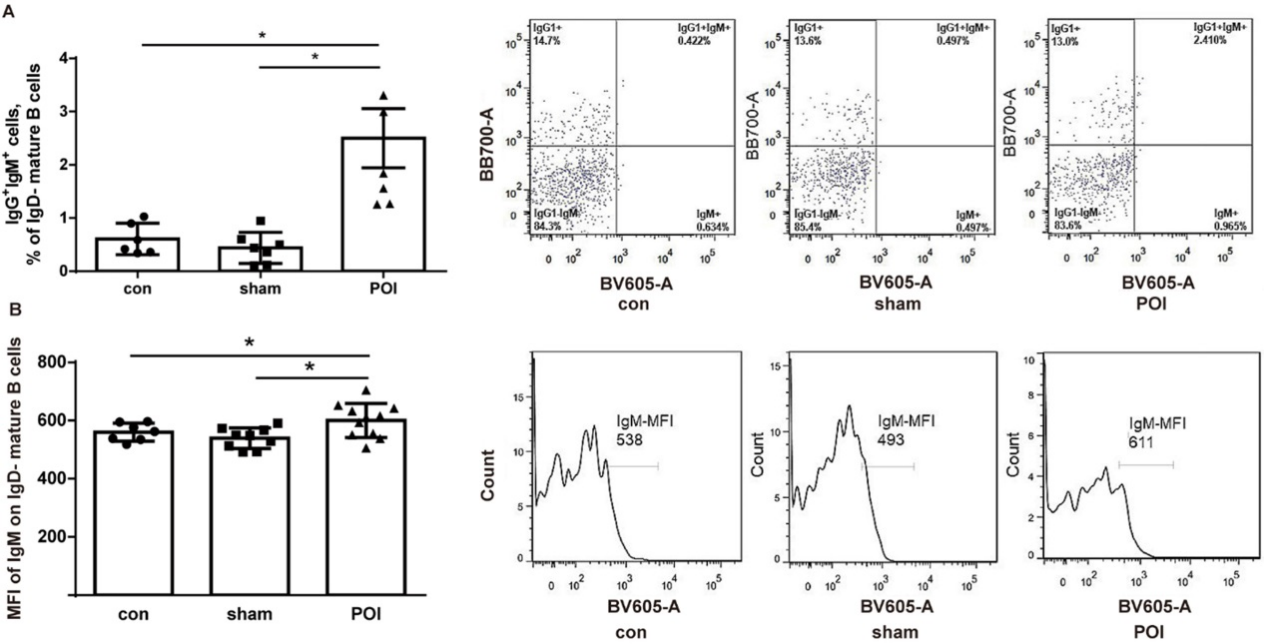
POI induces the Th2-dependent activation of memory B cells. (A) Memory B cells measured as described in Materials and Methods and presented as a bar graph (mean with SD), n=6-8 (per group), analyzed with the ordinary one-way ANOVA with the Tukey's post-test, * *p*<0.05 - left panel, and as a representative FACS picture - right panel. (B) Intensity of the surface IgM expression on memory B cells expressed in the mean fluorescence intensity (MFI), analyzed with the ordinary one-way ANOVA with the Tukey's post-test, * *p*<0.05 - left panel, and as a representative FACS histogram picture - right panel.

To assess whether POI influences or interacts with B cell development and activation, the amount of immature/transitional B cells in the PPs - defined as MHCII^-^ cells from the B220^+^/CD19^+^ gate - was investigated. We detected a low amount of immature/transitional B cells in PPs. The amount of these cells was significantly higher in the POI group (0.14 ± 0.08%), compared to control (0.01 ± 0.01%, *p* < 0.0001) or sham-operated animals (0.02 ± 0.01%, *p* < 0.0001). The rest of the B cells were mature ones (> 99%). These cells were gated further according to the surface expression of IgD, IgM, and IgG1 to discriminate between the memory and activated mature B cell phenotype. The amount of IgG1^+^IgM^+^ mature IgD^-^ cells which can be recognized as memory B cells was significantly higher in the POI group compared to the sham-operated mice or the control group (Figure 4A). It should be stressed that the expression of IgM by mean fluorescence intensity (MFI) levels was significantly increased in the POI group compared to sham-operated animals (Figure 4B). These findings reflect a higher activation state of memory B cells in POI.

**Figure 5:**
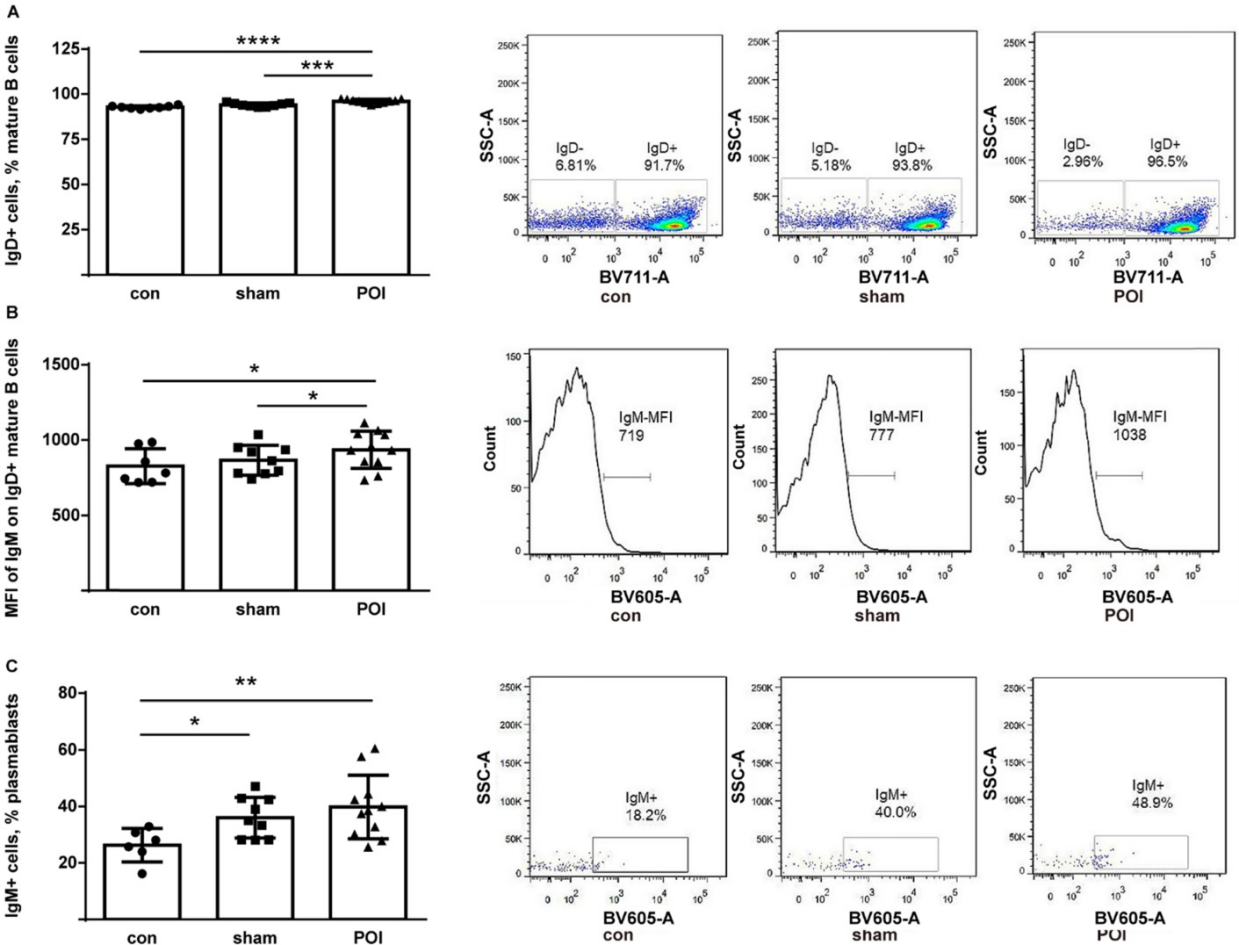
POI induces the accumulation of activated non-memory B cells. (A) Non-memory B cells were measured as described in Materials and Methods and presented as a bar graph (mean with SD), n=6-8 (per group). (B) The intensity of the surface IgM expression on non-memory B cells expressed in MFI. (C) Measurement of IgM^+^ plasmablasts as described in Material and Methods and presented as a bar graph (mean with SD), n=6-8 (per group). Data were analyzed with the ordinary one-way ANOVA with the Tukey's post-test, * *p*<0.05, ** *p*<0.01, *** *p*<0.001 and **** p<0.0001- left panel, and as a representative FACS picture - right panel.

The IgD^+^ compartment of mature B cells consists of follicular and activated non-memory cells. While all groups of mice possessed high amounts of these cells, POI mice showed the highest proportion of IgD^+^ mature B cells (Figure 5A). Besides, the high amount of these cells trended to correlate with worse motility among mice within the POI group (*r*=0.55, *p>0.0*5 but not in the group of control mice (data not shown). Furthermore, IgM expression was measured on the surface of IgD^+^ mature B cells and found to be significantly increased when compared to controls (Figure 5B). Thus, POI appears to be leading to an accumulation of activated non-memory B cells in PPs.

**Figure 6:**
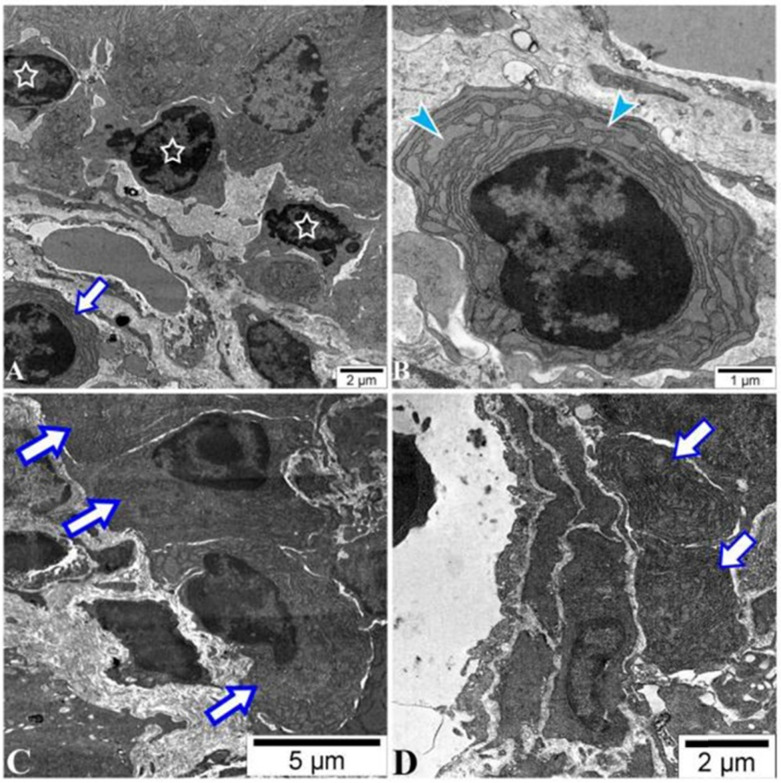
POI leads to the accumulation of plasma cells in the ileum. Histotopography and ultrastructural characteristics of plasma cells in ileum after 48 h of the POI induction assessed by electron microscopy. (A) Lymphocytes (asterisks) with a fragment of plasma cells (arrow) placed in the intracellular space of enterocytes. Plasma cells (B) placed in the villi (C) or nearby of a lymphatic vessel (D) in the submucosa of the ileum.

There was a low amount of plasmablasts in PPs of all mice with no difference between groups by FACS analysis (data not shown). The IgM surface expression on the plasmablasts was higher in the sham and POI group (Figure 5C), respectively, indicating an influence of surgery on this group of B cells. Of note, plasma cells were found in the intracellular space of enterocytes in the ileum of the POI mice by electron microscopy. These cells are characterized by extended granular endoplasmic reticulum filled with small granules indicating the active production of antibodies (Figure 6). The regulatory B cells defined as CD5^+^CD1^+^ population of B cells were virtually absent in the PPs of all mice tested (data not shown).

**Figure 7:**
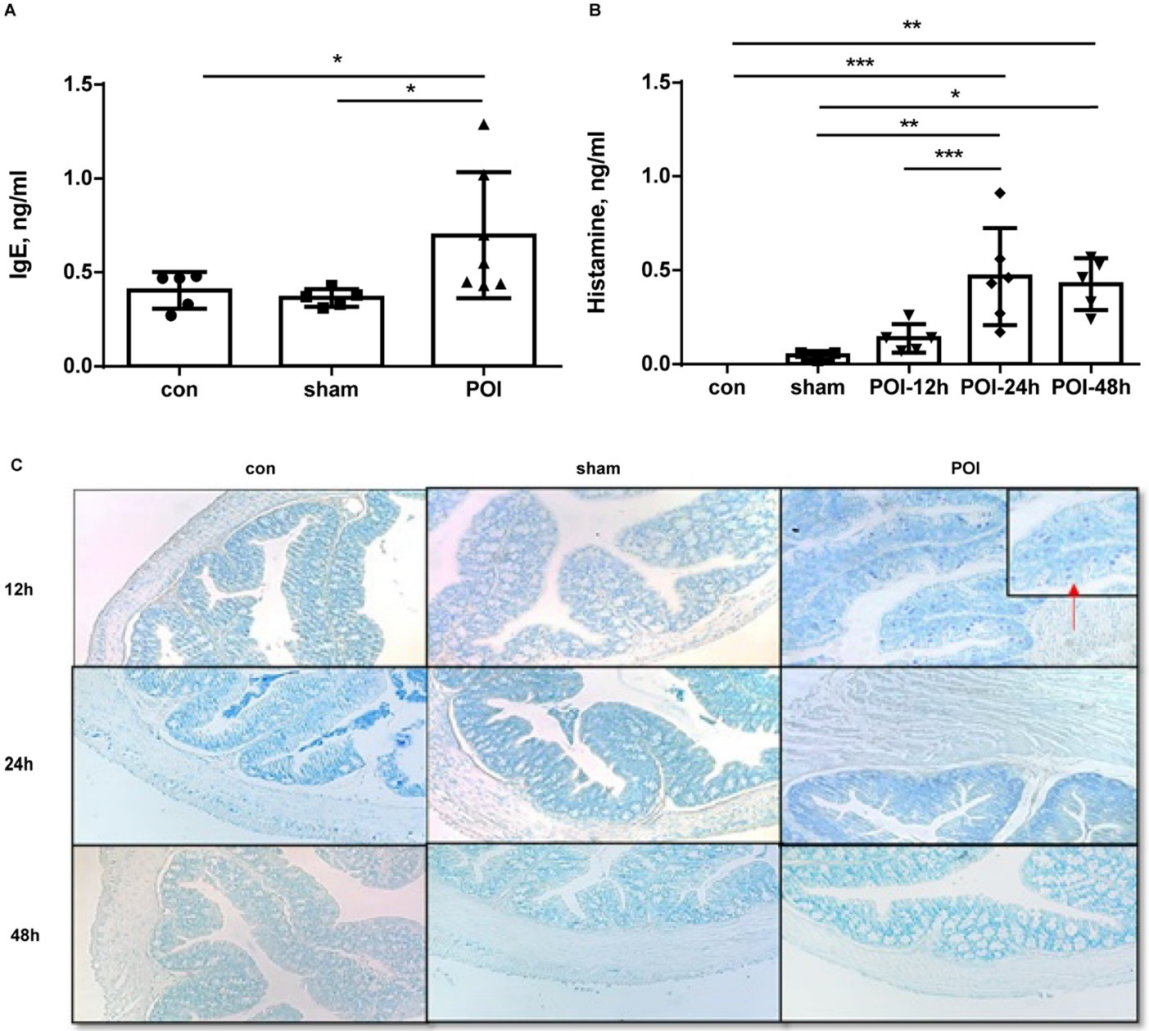
POI induces IgE secretion and activation of mast cells. (A) IgE plasma level measured as described in Material and Methods and presented as a bar graph (mean with SD), n=5-6 (per group), analyzed with the ordinary one-way ANOVA with the Tukey's post-test, * *p*<0.05. (B) histamine plasma level measured as described in Material and Methods and presented as a bar graph (mean with SD), n=5-6 (per group), analyzed with the ordinary one-way ANOVA with the Tukey's post-test, * *p*<0.05, ** *p*<0.01 and *** *p*<0.001. (C) Presence of mast cells, stained in colon slices as mentioned in the appropriate section.

Since the Th2 immune response seems to be involved in the POI pathogenesis through (memory) B cell activation, we investigated which cells are functioning as counterparts in this disease. Therefore, we measured soluble IgE in the plasma of mice. The levels of IgE were significantly higher in the POI group compared to both groups 24 h after POI induction (Figure 7A). The increase in IgE levels started at 12 h and reached a plateau at approximately 48 h after surgery (data not shown). High amounts of plasma IgE were associated with better intestinal motility among mice in the POI group (*r*=0.78, *p*<0.01) but not in the sham- or control group (data not shown). Based on the fact that soluble IgE activates mast cells, we further investigated the distribution of these immune cells in mouse colon slides. Of note, mast cells were solely detected in the colon from the POI mice after 12 h of surgery (Figure 7C). Because activated mast cells secrete histamine, we measured histamine levels in the plasma of mice. We found an increase in the histamine plasma concentration in POI mice, reaching a peak 24 h after POI induction (Figure 7B).

### POI-induced ROS regulate Th2-polarisation through transcription factors HMGB1, NF-κB and p38

**Figure 8:**
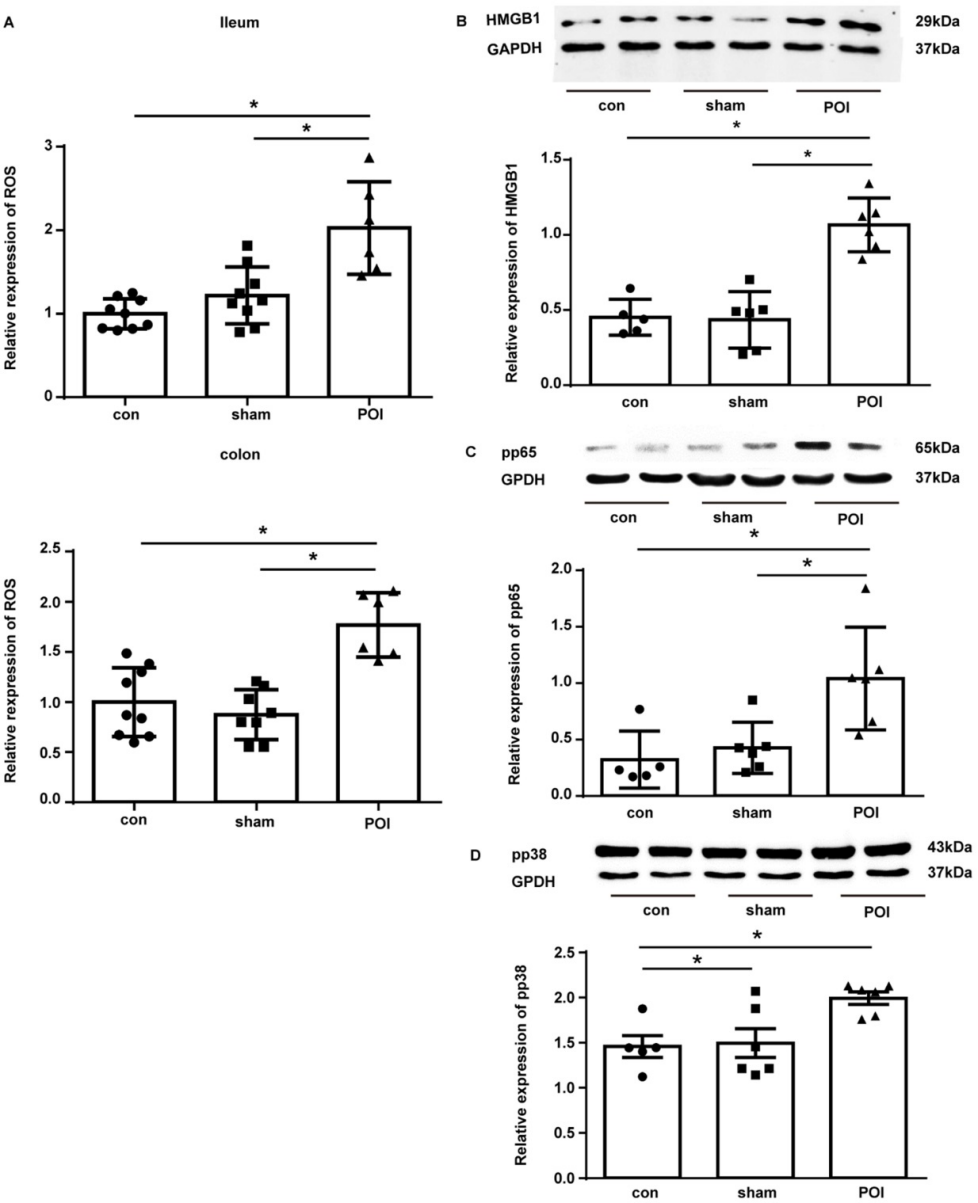
POI-induced ROS regulates expression of the Th2-polarisation transcription factor HMGB1, pp65 and pp38. (A) ROS levels measured as described in Materials and Methods and presented as a bar graph (mean with SD), n=6-9 (per group), analyzed with the ordinary one-way ANOVA with Tukey's post-test, * *p*<0.05. Analysis of HMGB (B), pp65 (C) and pp38 (D) expression in the colon with Western blot (upper panel) and densitometry of bands (lower upper), n=5-6 (two samples from each group are presented) analyzed with the ordinary one-way ANOVA with the Tukey's post-test, * *p*<0.05.

To further investigate the molecular mechanisms driving the Th2 immune response in POI, we looked at ROS which can lead to a shift between the Th1 and Th2 immune balance. Therefore, we assessed ROS levels in the ileum and colon. POI was associated with an accumulation of ROS in the ileum and colon beginning 12 h after surgery, whereas there was no such an increase noted in sham-operated animals (Figure 8A). It should be stressed that higher ROS levels correlated with decreased intestinal motility of mice in the POI group (*r*=-0.94, *p*<0.05) for ROS in the ileum, and for ROS in the colon (*r*=-1, *p*<0.05), but not in control or sham-operated animals. This data emphasizes the role of ROS in the pathogenesis of POI.

As High Mobility Group Box 1 (HMGB1) is responsible for the induction of Th2 polarization and its expression is under the regulation of ROS, we investigated the expression of this protein in the tissues of our experimental mice. Indeed, in the colon tissues collected from POI mice, there was a marked up-regulation of the HMGB1 expression compared to control and sham-operated animals (Figure 8B).

We further investigated the activation of NF-κB as an important regulator of MCP-1 and IL-10 expression by measuring the phosphorylated form of its subunit p65 (pp65) by Western blot. Figure 8C shows that pp65 was enriched in the colon of POI mice. Because ROS impact MCP-1 and IL-10 expression via up-regulation of p38, we measured the phosphorylated (activated) form of p38 (pp38). Western blotting showed an increased amount of pp38 in the colon of POI mice (Figure 8D). Hence, HMGB1, NF-κB and p38 represent potentially important transcription factors involved in the pathogenesis of POI by inducing Th2 polarization.

## Discussion

Although POI represents a well-known and frequent complication after surgery, the pathogenesis of POI remains elusive. This in turn drives the ongoing search for effective therapeutic approaches. Thus, new preclinical and clinical studies on POI are urgently needed for establishing effective prevention and treatment options. Inflammation has been demonstrated to play a key role in POI, manifesting itself in the development of a Th1 immune response 3, 12. Herein, we have presented evidence that Th2 immune response also appears to be involved in the pathogenesis of POI. We have confirmed the involvement of systemic inflammatory response in our experimental murine model of POI. IL-10 as well as MCP-1 are both known to be involved in the polarization of Th0 into Th2 lymphocytes 13,14, and indeed, lymphocytes of PPs were able to produce IL-4 leading to a significant increase in the intracellular concentration of the Th2-related cytokine in POI. In line with earlier data from ours and other working groups 7, 15, 16, we have found that the levels of inflammatory mediators such as IL-2, IL-10 and MCP-1 were increased in the blood of the POI mice, indicating a possible interaction of both Th1 and Th2 immune response. As immune cells produce both pro-inflammatory cytokines as well as anti-inflammatory cytokines, we tested other cytokines such as IL-6 and IFN-γ but there was no significant difference between the sham and the POI group. In contrast, the levels of IL-10 and MCP-1 in the sham and POI group were significantly different. Of note, IL-10 levels are known to be increased in POI patients as well as in POI animals; however, it is still debatable whether the role of IL-10 might be salutary in this setting 16, 17.

The cytokines MCP-1 and IL-10 are produced by monocytes, macrophages, and dendritic cells. In general, they are known to recruit lymphocytes to the inflammation site, inhibit IFN-γ production and promote IL-4 production by T cells. Furthermore, they promote the polarization of Th0 cells into the Th2 direction 18-20. In POI, we found an accumulation of monocytes in PPs under POI conditions. In a previously published study, immune cells from the intestinal muscular layer were isolated and analyzed 3. However, we have looked at immune cells derived from PPs as the primary intestinal immune sensors, which function to induce immune tolerance or defense against pathogens and to ensure intestinal homeostasis at an early stage 21, 22. Consistent with the findings from Engel et al. 3, we saw an increase in Th1 cells in POI, even if derived from PPs. However, in contrast to Engel et al., CD4^+^ lymphocytes from PPs were able to produce IL-4, indicating a possible involvement of Th2 immune response in POI. This substantial difference to other earlier studies could be explained by considering the sources of immune cells studied.

Mast cells play a prominent role in the development of POI 23-25. The activation of mast cells requires the involvement of IgE, which may mainly be derived also from active B cells, and, can be induced through stimulation from cytokines produced by Th2 cells. Based on our new data, we suggest that B cells - especially activated memory and non-memory B cells - activate mast cells through soluble IgE in experimental POI.

It is generally accepted that intestinal manipulation leads to degranulation of mast cells. This is followed by an increase in mast cell proteases and histamine release, resulting in reduced gastrointestinal motility 23. Based on this mechanism, we proposed that IgE produced by B cells under POI conditions can also activate mast cells. Therefore, we analyzed systemic IgE levels in mice plasma. Surprisingly, a high amount of IgE in POI mice correlated positively with better intestinal motility. Hence, a negative correlation between systemic and local concentrations of IgE could be existing. In addition, the fact that mast cells were only present soon after POI induction could signal mast cell degranulation. The elevated histamine levels which were found 24 h and 48 h after surgery or POI induction would also add weight to this.

Trying to explore the molecular mechanism behind the findings above, we paid attention to the fact that ROS levels were found to be significantly increased in POI. ROS are known to be involved in the regulation of Th0 polarization in the Th2 direction, through the transcription factors HMBG1, NF-κB and p38 13, 26-32. For example, MCP-1 and IL-10 expressions are under the regulation of the transcription factor NF-κB whose expression is also regulated by ROS 33-36. Furthermore, there is data showing that ROS can directly activate mast cells themselves 36. In line with this, we demonstrated an increase in intestinal ROS levels under POI conditions, as well as an up-regulation of the transcription factors HMGB1, NF-κB and p38.

It should be mentioned that next to mast cells, basophils as antigen-presenting cells also preferentially induce Th2 immune response 37. And similar to mast cells, basophils can readily produce histamine and IL-4. In addition, basophils derived IL-4 was shown to contribute to the tissue infiltration of eosinophil cells 37-39. Another study clearly indicated that regulatory Treg promotes the activation of basophils, leading to an enhanced Th2 environment 40. There are other models of POI such as the LPS-induced POI model. LPS is another way to induce ileus but is based on different molecular and cellular immune mechanisms 41. In the current work, we did not look at these mechanisms. Therefore, it is important to further explore whether Th2 immunity could be also involved in the development of LPS induced motility promoting the understanding of postoperative ileus. In addition, the level of Th2 cytokines and immune cells within the gut need to be explored in our future work. This may offer new targets for a perioperative medication to prevent or ameliorate the development of POI.

In summary, our findings indicate an alternative way of mast cell activation in POI. Intestinal manipulation leads to an increase in the production of ROS. These highly active molecules can activate mast cells themselves and shift Th0 cells towards the Th2 direction through up-regulation of HMBG1, NF-κB, and p38. Th2 cytokines influence B cells to secrete IgE which finally activates mast cells to degranulate and release histamine. This non-conventional way of mast cell activation in POI looks like an allergy-like reaction. Based on these findings, it is quite evident that routinely used prokinetic agents do not function in this setting. Accordingly, new treatment options for POI such as strategies to diminish ROS production should be tested.

## Supplementary Material

Supplementary figures.

## Abbreviations

POI: postoperative ileus
ERAS: enhanced recovery after surgery
ROS: reactive oxygen species
DCFDA: 2,7-dichlorfluorescein diacetate
WT: wild type
PPs: peyer's patches
FACS: Flow Cytometry
SD: standard deviation
pp38: Phospho-p38
