# Supplementary material for: Experimental postoperative ileus: is Th2 immune response involved?
Source: Int J Med Sci. 2021 Jun 16;18(13):3014–25. doi: 10.7150/ijms.59354 (PMC8241774; doi:10.7150/ijms.59354)
Supplement: Supplementary file 1 — Supplementary figures. [file ijmsv18p3014s1.pdf]

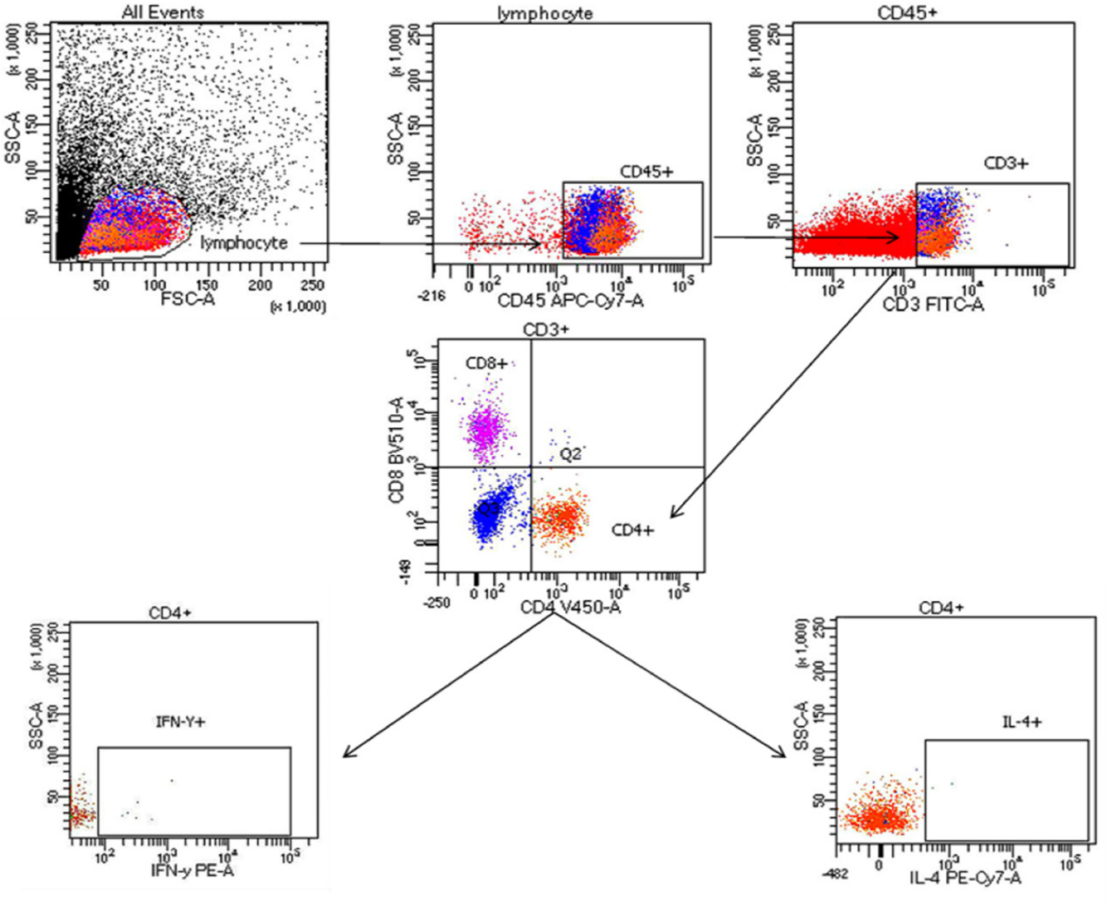

Figure S1. FACS gating strategy of IFN+ cells and IL-4+ cells

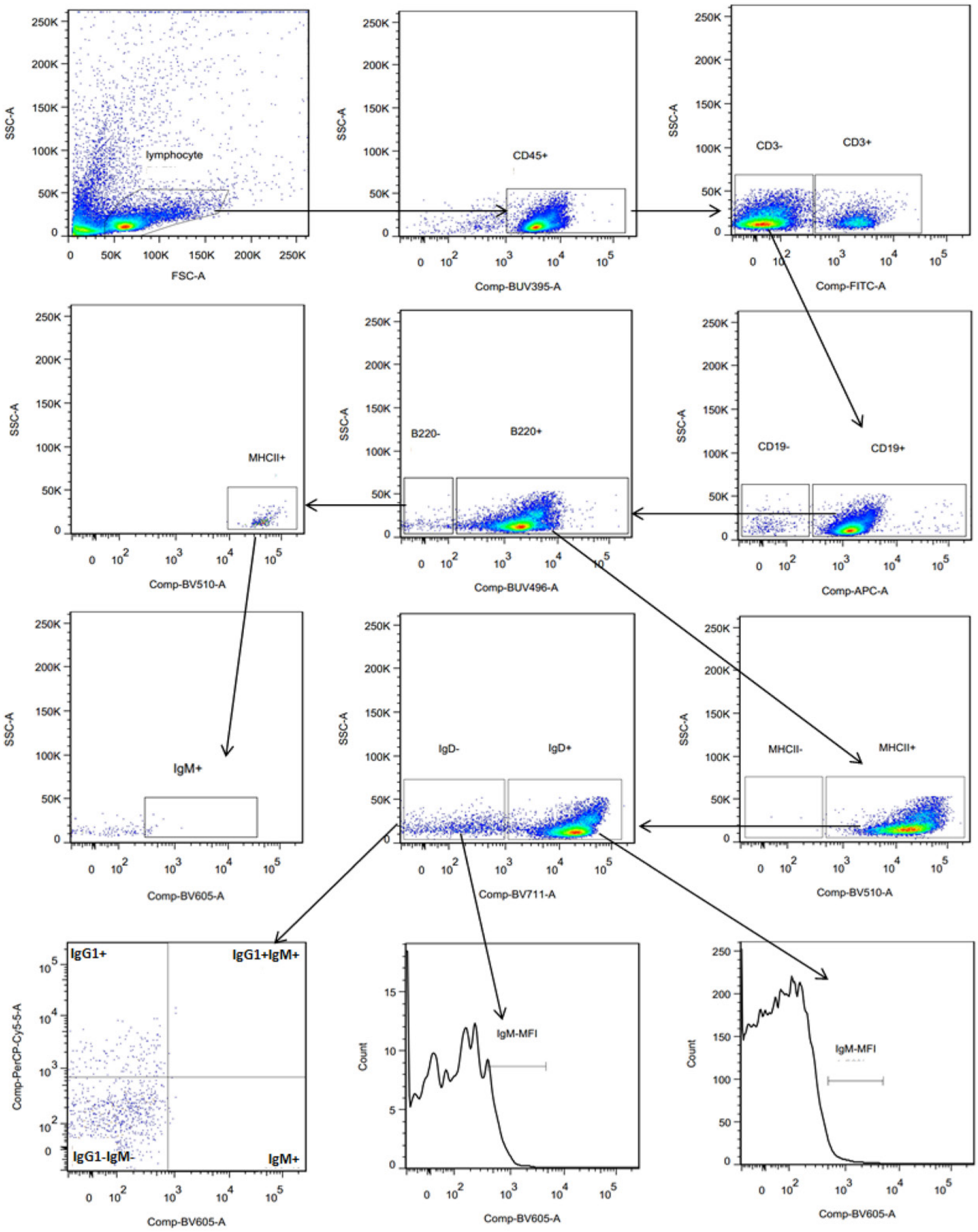

Figure S2. FACS gating strategy of B-cell subpopulation.
